# Paediatric pulmonary disease—are we diagnosing it right?

**DOI:** 10.3389/fped.2024.1370687

**Published:** 2024-04-10

**Authors:** Priya Rajendran, Silla Varghese Thomas, Sarath Balaji, Elilarasi Selladurai, Ganesh Jayachandran, Aravind Malayappan, Adhin Bhaskar, Sivaraman Palanisamy, Thirumalani Ramamoorthy, Sindhu Hasini, Syed Hissar

**Affiliations:** ^1^ ICMR—National Institute for Research in TB, Chennai, India; ^2^ Institute of Child Health, Chennai, India; ^3^ Government Stanley Medical College and Hospital, Chennai, India

**Keywords:** tuberculosis, pneumonia, children, coinfection, multiplex real-time PCR, diagnosis

## Abstract

**Background:**

It has been reported that differential diagnosis of bacterial or viral pneumonia and tuberculosis (TB) in infants and young children is complex. This could be due to the difficulty in microbiological confirmation in this age group. In this study, we aimed to assess the utility of a real-time multiplex PCR for diagnosis of respiratory pathogens in children with pulmonary TB.

**Methods:**

A total of 185 respiratory samples [bronchoalveolar lavage (15), gastric aspirates (98), induced sputum (21), and sputum (51)] from children aged 3–12 years, attending tertiary care hospitals, Chennai, India, were included in the study. The samples were processed by N acetyl L cysteine (NALC) NAOH treatment and subjected to microbiological investigations for *Mycobacterium tuberculosis* (MTB) diagnosis that involved smear microscopy, Xpert® MTB/RIF testing, and liquid culture. In addition, DNA extraction from the processed sputum was carried out and was subjected to a multiplex real-time PCR comprising a panel of bacterial and fungal pathogens.

**Results:**

Out of the 185 samples tested, a total of 20 samples were positive for MTB by either one or more identification methods (smear, culture, and GeneXpert). Out of these 20 MTB-positive samples, 15 were positive for one or more bacterial or fungal pathogens, with different cycle threshold values. Among patients with negative MTB test results (*n* = 165), 145 (87%) tested positive for one or more than one bacterial or fungal pathogens.

**Conclusion:**

The results suggest that tuberculosis could coexist with other respiratory pathogens causing pneumonia. However, a large-scale prospective study from different geographical settings that uses such simultaneous detection methods for diagnosis of childhood tuberculosis and pneumonia will help in assessing the utility of these tests in rapid diagnosis of respiratory infections.

## Introduction

Children and young adolescents aged under 15 years represent about 11% of the total population globally, where 1.1 million of them fall ill with tuberculosis (TB) every year, and more than 225,000 of them lose their lives (1). In addition to TB, pneumonia caused by other respiratory pathogens serves as a major cause of morbidity and mortality worldwide, especially in Southeast Asia and sub-Saharan Africa, collectively enduring the largest burden of more than half the worldwide total cases of pneumonia in children (2–4). According to the 2023 India TB report, the prevalence of TB in Tamil Nadu was 180 per 10,000 people out of which 8.2% were of paediatric population. In recent years, non-resolving pneumonia has posed a major setback in treating children because of its clinical manifestations, persistent symptoms, and the slow pace of radiographic resolution (5). Fein et al. combined clinical and radiologic indices and defined non-resolving pneumonia as slow resolution of radiologic infiltrates or persistent clinical symptoms despite appropriate antibiotic therapy (6). Management of this non-responding disease requires a complete microbiological investigation, with a broad spectrum of aetiology to cover unusual pathogens as well. For example, children infected with *Mycobacterium tuberculosis* (MTB) present with similar clinical manifestations as of community-acquired pneumonia (CAP) caused by other bacteria/viruses and termed as acute tuberculous pneumonia (TP). While pulmonary TB is characterized by the formation of lung injury and tubercles, TB caused by MTB is characterized by acute respiratory infection, including dry cough, fever, and chest pain. Hence, misdiagnosis of TB as CAP can lead to wrong/delayed treatment and patient management. Similarly, instances of CAP with aetiology other than MTB being diagnosed as TB are also reported.

In addition, although TB can directly cause severe pneumonia and disseminated disease, children are likely to present to hospital with diverse aetiology complicating underlying pulmonary TB. Consequently, there is a growing awareness that children have a high burden of TB-related disease that is often not reported as such (7). The concept that TB might increase susceptibility to secondary bacterial pneumonia in young children is being reported by a range of studies (8–11). These studies reported that the proportion of pneumonia cases diagnosed with co-infected TB ranged from 1% to 23%. The autopsy studies provide additional data on the contribution of TB to pneumonia-related deaths in children. The contribution ranged from 4% to 20% in children who died from respiratory disease in these settings with very high TB incidence rates (12). Moreover, a recent systematic review documented that, since clinical and radiological features of TB disease are indistinguishable from other causes of pneumonia in children, their empirical treatment with antibiotics makes the follow-up and treatment for TB difficult at later stages (13). The interpretations of such findings from various studies implicate that there is a need for an extensive study on the association of TB with CAP in children by strengthening the microbiological confirmation methods in this age group, especially in resource-restricted TB endemic settings (14). This will in turn help to better understand the contribution of TB to childhood CAP and to improve clinical management. In this study, we hypothesized that simultaneous diagnosis of TB and CAP will depict the factual representation of the aetiology. Hence, we aimed to detect the aetiology of CAP in children with presumptive TB using a comprehensive diagnostic approach.

## Materials and methods

### Study setting and sample collection

The cross-sectional pilot study was conducted at Chennai, India, where the patients were recruited from the Institute of Child Health and Government Stanley Medical College and Hospital, and laboratory procedures were carried out at the Indian Council of Medical Research (ICMR)—National Institute for Research in TB (NIRT). A total of 185 children between 3 and 12 years of age were included in the study. Of these, 121 children with clinical signs and symptoms suggestive of TB and previously treated with antibiotics adequately were included into the presumptive TB group and 64 children with acute pulmonary infection with fever, cough, malaise, and/or dyspnoea and yet to be treated with antibiotics were included into the pneumonia group. Grouping the children into the presumptive TB or presumptive pneumonia groups entirely depended on the clinician's decision based on the WHO definition of pneumonia and TB, chest x-ray findings, and clinical symptoms. Patients not meeting the criteria (not willing to give consent or adequate samples) were excluded from the study.

Adequate quantities (3–5 ml) of expectorated sputum were collected from children in transparent, wide-mouthed, and leak-proof containers and were properly labelled. If a child was not able to produce sputum and if the clinician suggested collection of bronchial wash or gastric aspirate, the same was collected by trained personnel. Bronchoalveolar lavage was collected from 15 patients, gastric aspirates from 98, induced sputum from 21, and sputum from 51 children aged 3–12 years, attending tertiary care hospitals in Chennai, India. Clinical details of the children along with their or family's previous TB history, and Tuberculin Sensitivity Test (TST) results were also collected.

### Laboratory testing

The respiratory samples were processed by N acetyl L cysteine (NALC) NaOH and subjected to further processing for smear, culture, and Xpert testing. The smears were stained with Auramine and Gram's stain, and were observed under fluorescent and light microscopy, respectively, to look for acid-fast MTB bacilli and other bacterial pathogens. The smear grading of acid-fast bacilli was done according to WHO guidelines (1). The processed samples were subjected to both liquid [Mycobacteria Growth Indicator Tube (MGIT)] and solid culture [Lowenstein–Jensen (LJ)]. In addition, Xpert MTB/rifampicin (RIF) testing was done to detect MTB and its RIF resistance.

### DNA extraction

A part of the processed sample was subjected to DNA extraction by cetyltrimethylammonium bromide (CTAB) method (15). Briefly, the sample was mixed with 30 µl of 10% SDS and 3 µl of 20 µg/ml proteinase K and incubated for 1 h at 56°C. To this mixture, 100 µl of 5 M NaCl and 80 µl CTAB/NaCl solutions were added and incubated for 10 min at 65°C. Equal volume of chloroform was added and centrifuged at maximum speed, and the supernatant was transferred. The supernatant was used for the multiplex real-time PCR.

### Multiplex real-time PCR

Multiplex real-time PCR was done using the QIAGEN—Microbial DNA qPCR Array Kit for the detection of a panel of bacterial and fungal pathogens on extracted DNA. The list of the bacteria that were used for the detection included *Streptococcus pneumoniae, Haemophilus influenzae, Moraxella catarrhalis, Staphylococcus aureus, Escherichia/Shigella* spp, *Klebsiella pneumoniae, Pseudomonas aeruginosa, Acinetobacter baumannii*, *Mycoplasma pneumoniae, Bordetella* spp, *Chlamydia pneumoniae, Legionella pneumophila, Mycobacterium* spp, *Mycobacterium avium, Mycobacterium kansasii, Mycobacterium tuberculosis*, *Nocardia* spp, *Aspergillus flavus, Aspergillus fumigatus, Pneumocystis jirovecii,* PANA—Pan Aspergillus/Penicillium, PANB1—Pan Bacteria1, and PANB3—Pan Bacteria 3. PANB1 include Actinobacteria, Bacteroidetes, Euryarchaeota, Firmicutes, Fusobacteria, Proteobacteria, and Tenericutes. In addition, Pan Bacteria 3 detected species within the phylum Spirochaetes (Table 1). The interpretation of the real-time PCR results was done as per the Kit protocol (16). A CT value of 10–20 means high load of bacteria, which indicates that the microorganism could be a pathogen rather than commensal. A CT value of 20–30 means medium load and 30–40 means low load, indicating commensal rather than pathogen. A total of four samples can be tested in a single run.

**Table 1:** Panel of organisms detected by multiplex real-time PCR.

	Sample 1			Sample 2			Sample 3			Sample 4		
	1	2	3	4	5	6	7	8	9	10	11	12
A	SP	MP	NO. S	SP	MP	NO. S	SP	MP	NO. S	SP	MP	NO. S
B	HI	BS	AFL	HI	BS	AFL	HI	BS	AFL	HI	BS	AFL
C	MC	CP	AFU	MC	CP	AFU	MC	CP	AFU	MC	CP	AFU
D	SA	LP	PJ	SA	LP	PJ	SA	LP	PJ	SA	LP	PJ
E	ES	MY	PF	ES	MY	PF	ES	MY	PF	ES	MY	PF
F	KP	MA	PANB1	KP	MA	PANB1	KP	MA	PANB1	KP	MA	PANB1
G	PA	MK	PANB3	PA	MK	PANB3	PA	MK	PANB3	PA	MK	PANB3
H	AB	MY	PPC	AB	MY	PPC	AB	MY	PPC	AB	MY	PPC

SP, *Streptococcus pneumoniae;* HI, *Haemophilus influenzae;* MC, *Moraxella catarrhalis;* SA, *Staphylococcus aureus;* ES, *Escherichia/Shigella* spp; KP, *Klebsiella pneumoniae;* PA, *Pseudomonas aeruginosa;* AB, *Acinetobacter baumannii*; MP, *Mycoplasma pneumoniae;* BS, *Bordetella* spp; CP, *Chlamydia pneumoniae*; LP, *Legionella pneumophila;* MY, *Mycobacterium* spp; MA, *Mycobacterium avium;* MK, *Mycobacterium kansasii;* MT, *Mycobacterium TB*; NO.S, *Nocardia* spp; AFL, *Aspergillus flavus;* AFU, *Aspergillus fumigatus;* PJ, *Pneumocystis jirovecii;* PANA, Pan Aspergillus/Penicillium; PANB1, Pan Bacteria1; PANB3, Pan Bacteria 3.

## Results

**Figure 1:**
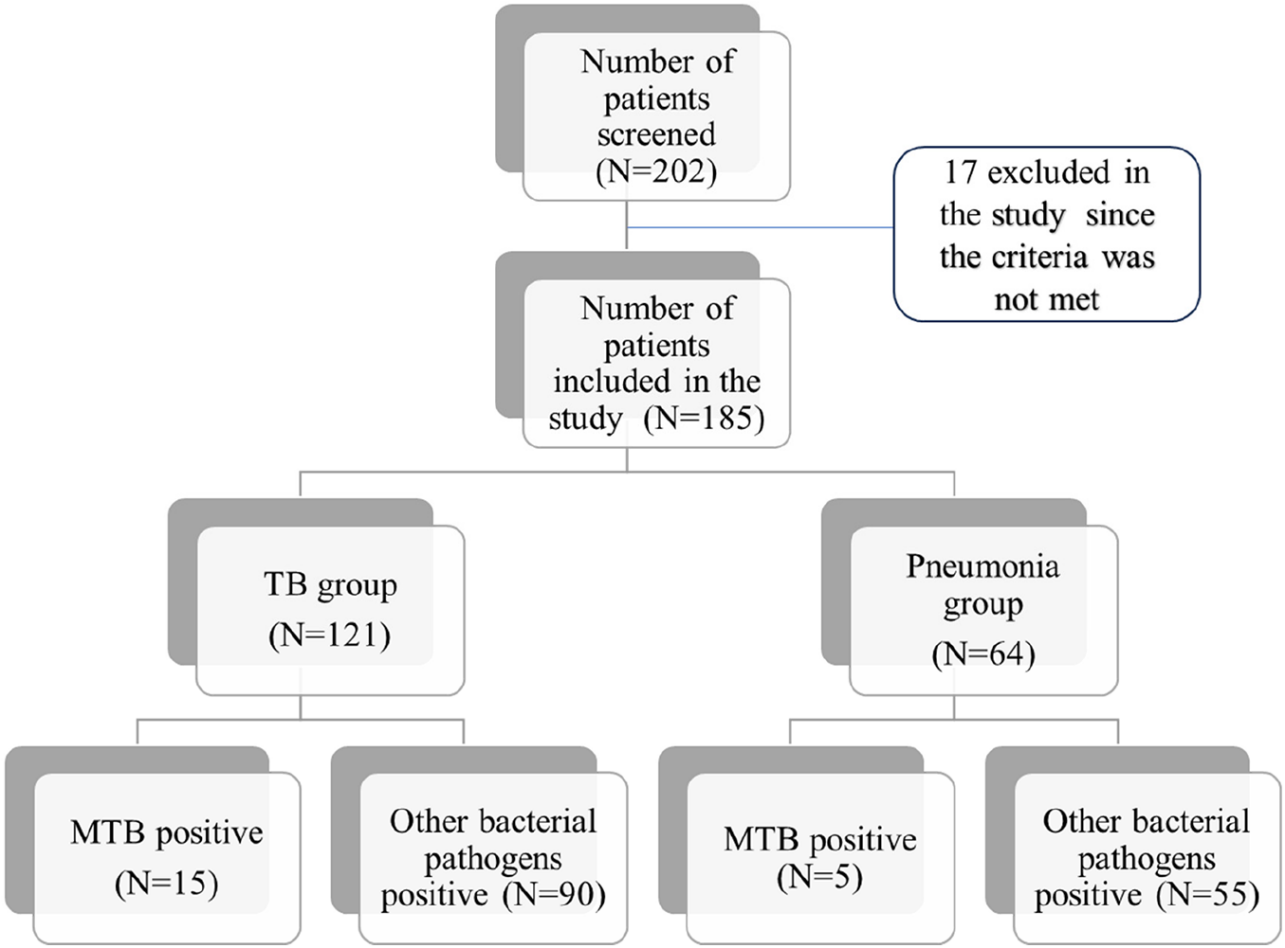
Flow chart of patients included in different groups.

A total of 202 children with symptoms suggestive of pneumonia or presumptive TB were recruited for the study. Of these, 185 patients were included in the study, with 121 in the TB group and 64 in the pneumonia group. The interquartile range (IQR) of the age of the patients ranged from 7 to 10. A total of 165 patients had aetiological agents of pneumonia with single or multiple organisms isolated from each patient (Figure 1). During the clinical presentation at the hospital, cough and fever were the most common symptoms found in 126 patients (67%) followed by tiredness in 74 (40%), weight loss in 64 (34%), difficulty in breathing in 43 (23%), and chest pain in 22 (12%) patients.

### Distribution of bacterial pathogens in MTB-positive patients

**Figure 2:**
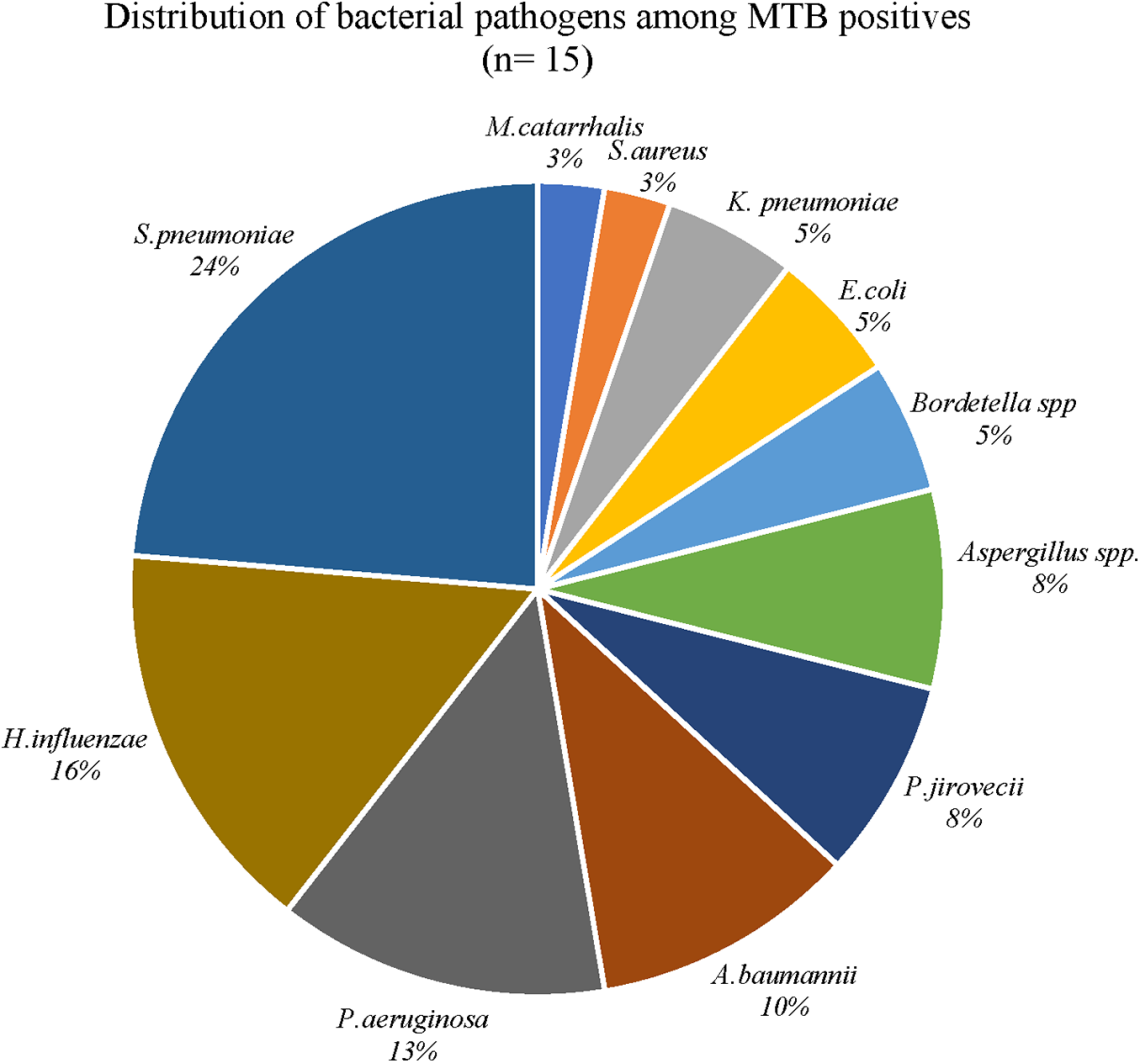
Distribution of bacterial pathogens among MTB positives in the TB group.

Out of these 185 children, 20 (11%) were positive for MTB by either one of the methods tested (microscopy/culture methods/Xpert Ultra). Out of these 20 MTB positives, 15 (75%) were found to be co-infected with one or more bacterial pathogens. Three out of the 15 co-infected children were from the pneumonia group and 12 from the TB group. Among the five MTB positives without any coinfection, two were from the pneumonia group and three from the TB group. When the distribution of other bacterial pathogens among the MTB-positive patients were explored, *S. pneumoniae* was the predominant pathogen in 9 patients and had cycle threshold (CT) values ranging from 20 to 32 and its association with TB was found to be significant by chi square test (*p* < 0.05). This was followed by *P. jirovecii,* identified in three patients with lowest CT value 18.33, *K. pneumoniae* in two patients (23.26), and *H. influenzae* in six patients (24.11). Other organisms identified had CT values ranging from 30 to 37 (Table 2 and Figure 2).

**Table 2:** Characteristics of patients positive for MTB*.*

S. No	Group	Age	TST	X-ray	Microbiology				Other bacteria identified	CT value
					Smear	LJ	MGIT	Xpert		
1	T	12	Not done	Normal	Neg	Neg	POS	Low	*S. pneumoniae* *H. influenzae* *S. aureus Bordetella sp.*	20.87 24.88 33.36 34.96
2	T	7	Not done	Abnormal	2+	Neg	Neg	Neg	*A. baumannii*	35.7
3	T	9	Positive	Abnormal	Neg	Neg	POS	Low	*E. coli*	33.87
4	P	10	Positive	Abnormal	Neg	Neg	POS	Neg	*A. fumigatus* *P. jirovecii*	33.28 21.68
5	T	7	Negative	Normal	Neg	Neg	POS	Neg	*S. pneumoniae* *H. influenzae*	26.48 37.64
6	T	8	Negative	Abnormal	1+	3+	POS	Medium	*S. pneumoniae* *H. influenzae* *P. aeruginosa* *K. pneumoniae* *A. baumannii*	32.55 35.32 30.23 23.26 35.32
7	T	5	Negative	Not done	Neg	1+	POS	Medium	0	
8	T	11	Negative	Abnormal	1+	1+	POS	Neg	0	
9	T	11	Positive	Abnormal	Neg	2col	POS	Medium	*S. pneumoniae* *A. flavus* *E. coli* *Bordetella*	30.1 38.65 37.85 35.02
10	T	11.5	Negative	Abnormal	Neg	Neg	Neg	Medium	*S. pneumoniae* *P. aeruginosa* *A. baumannii*	27.2 34.29 37.7
11	T	11	Not done	Not done	Neg	1col	Neg	Neg	*S. pneumoniae* *H. influenzae*	30.47 31.57
12	T	6	Not done	Normal	Neg	Neg	POS	Neg	*S. pneumoniae*	29.2
13	T	12	Positive	Abnormal	2+	2+	POS	Medium	0	
14	T	9	Not done	Not done	Neg	1+	POS	Medium	*S. pneumoniae* *H. influenzae* *M. catarrhalis* *P. aeruginosa*	27.44 33.75 30.73 34.66
15	P	9	Positive	Abnormal	Neg	1+	POS	Neg	*K. pneumoniae* *A. baumannii*	35.64 36.7
16	P	12	Not done	Abnormal	Neg	1col	POS	Medium	*P. aeruginosa*	35.56
17	T	3	Not done	Not done	Neg	Neg	Neg	Medium	*S. pneumoniae* *H. influenzae* *P. aeruginosa* *P. jirovecii*	30.59 32.99 33.24 35.26
18	P	2.5	Positive	Not done	Neg	Neg	Neg	Low	0	
19	T	8	Not done	Abnormal	Neg	2col	POS	Neg	*A. fumigatus* *P. jirovecii*	30 18.33
20	P	11	Not done	Not done	Neg	Neg	Neg	Low	0	

### Distribution of bacterial pathogens in MTB-negative patients

**Figure 3:**
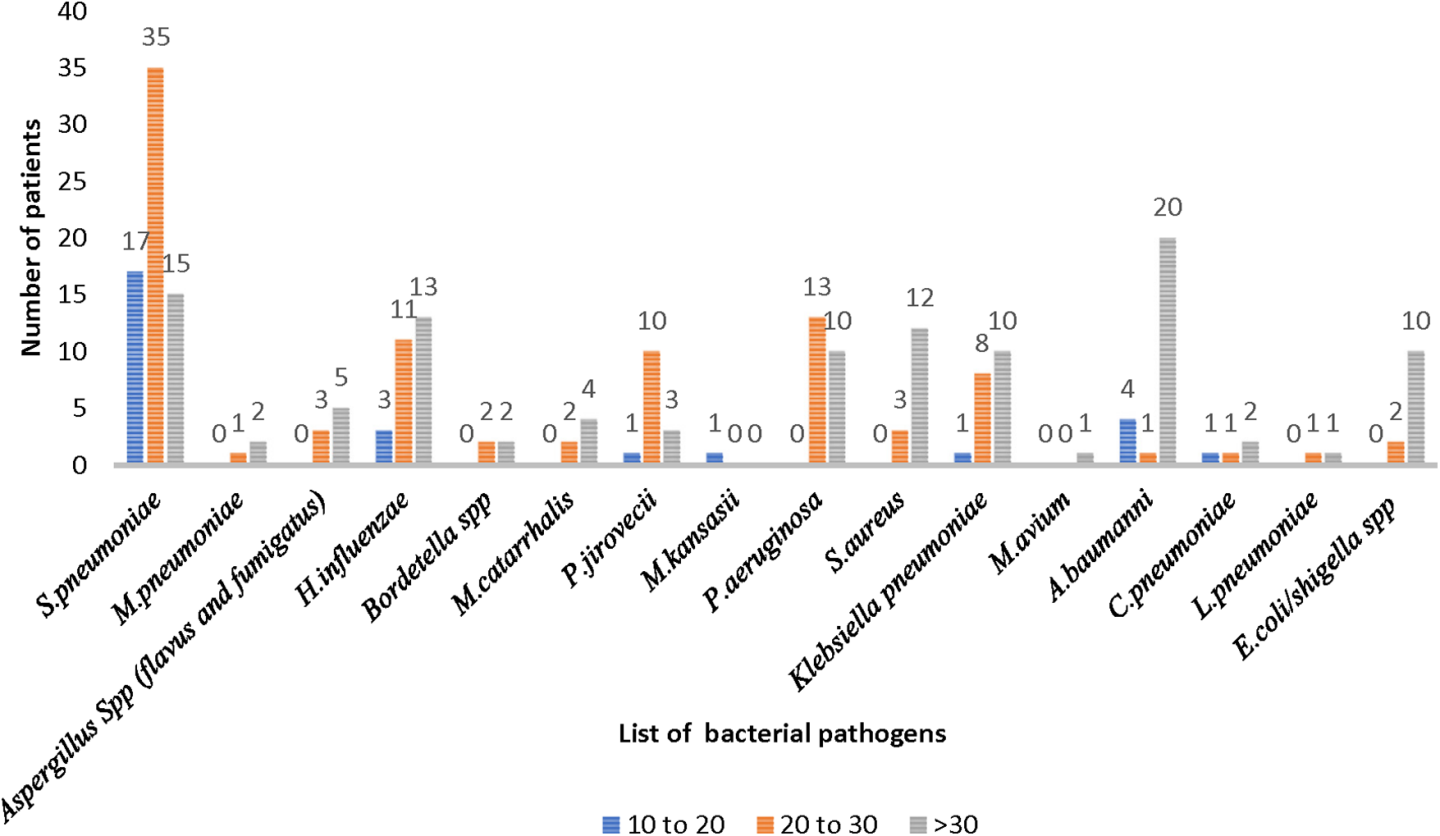
Distribution of bacterial pathogens causing pneumonia in the MTB-negative TB group (*n *= 90).

Among the MTB-negative patients (*n* = 165; 106 in the TB group and 59 in the pneumonia group), 145 (87%) were positive for one or more than one bacterial or fungal pathogens. Out of these 145, 90 (62%) were from the TB group and 55 (38%) from the pneumonia group. When the distribution of bacterial pathogens was explored among the patients in the MTB-negative TB group (*n* = 90), *S. pneumoniae* was the predominant pathogen (*n* = 67) with higher load in 17 patients (19%), medium load in 35 (38%) patients, and lower load in 15 patients (16%). The next predominant bacteria was *H. influenzae* (*n* = 27), with a higher load in 3 patients, medium load in 11 patients, and a low load in 13 patients. Other bacteria identified with different loads are depicted in Figure 3.

**Figure 4:**
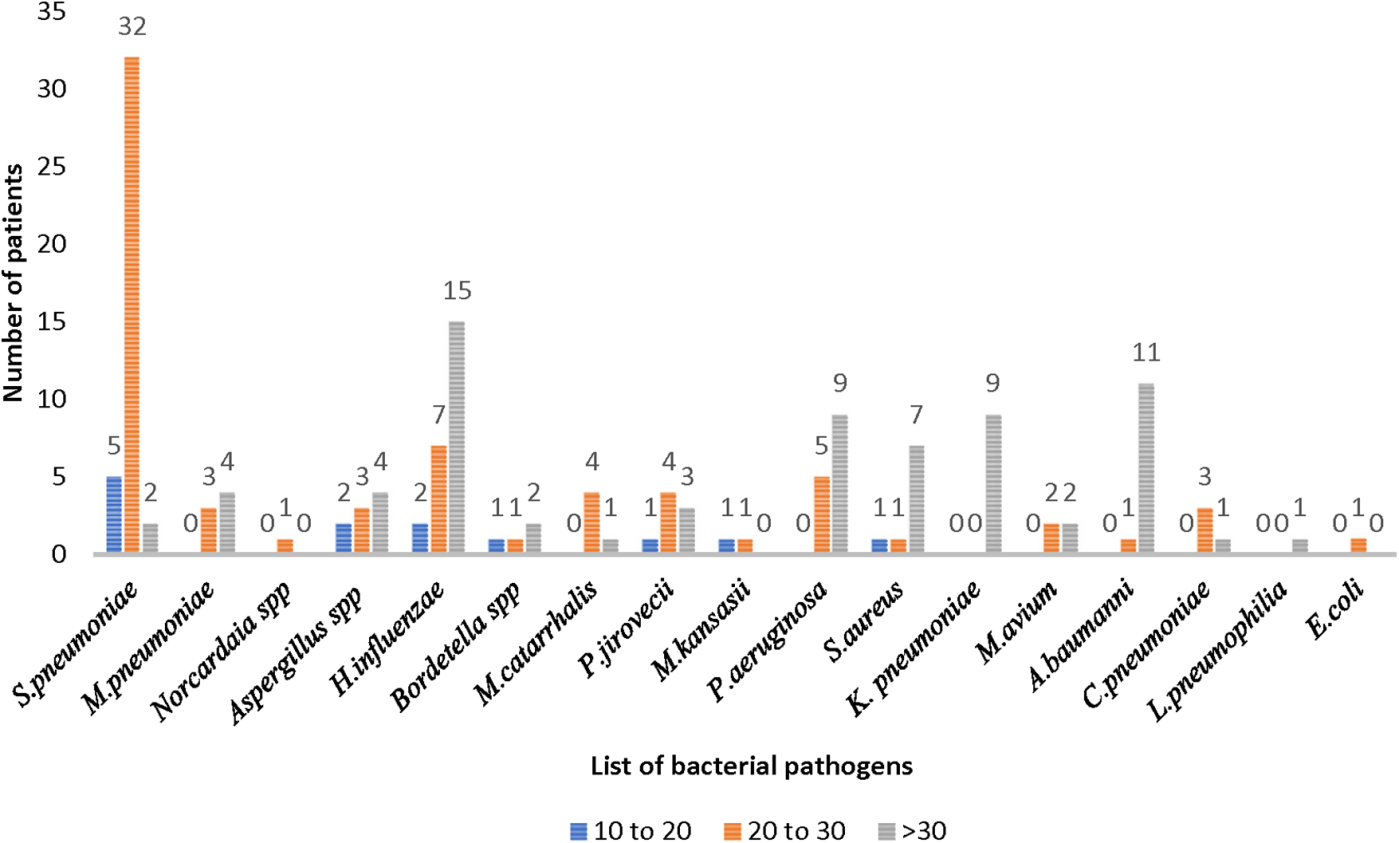
Distribution of bacterial pathogens causing pneumonia in the MTB-negative pneumonia group (*n *= 55).

Among the MTB-negative pneumonia group (*n* = 59), *S. pneumoniae* was identified in 39 patients, with 32 patients showing medium load, 5 patients, high load, and 2 patients, low load. Similar to the TB group, *H influenzae* was the second most common pathogen identified (*n* = 24) in 15 patients as low load, 7 patients, as medium load, and 2 patients as high load. Other bacteria identified with different loads are depicted in Figure 4.

The distribution of bacteria among the TB and the pneumonia group was found to be more than two in most of the patients with identification of up to seven in two patients from both the groups (Table 3).

**Table 3:** Number of patients in the pneumonia and TB groups harbouring multiple bacteria.

No. of bacteria	No. of patients pneumonia group	No. of patients TB group
2	15	24
3	10	14
4	10	12
5	6	6
6	1	6
7	2	2

### Clinical characteristics

During recruitment, chest x-ray findings were available for 125 out of 185 patients. Out of 125, 89 (71%) and 36 (29%) had abnormal and normal chest x-rays, respectively. The presence of infiltrates and/or consolidations was found in 28 patients (31%). Unilateral abnormalities were found in 30 patients and bilateral abnormalities were found in 11 patients. Among the patients with abnormal chest x-rays, 70 patients had one or more than one bacterium, 2 had MTB alone, 9 had both MTB and bacteria, and 8 patients had no organisms identified. However, there was no significant correlation between the organisms identified and chest x-ray findings. Of the 36 patients with normal chest x-ray, 28 had only bacterial pathogens, 3 had both TB and bacteria, while 5 of them had no organisms identified. Although fever and cough were common symptoms found in the screened population, the duration of fever and cough was more among patients with MTB and other bacteria with an average of 23 days, while patients diagnosed with other bacteria alone had an average of 17 days.

Out of the 185 patients, only 116 could be tested for TST and among them 48 (41%) were positive, of which 6 were positive for MTB, 33 were positive for other bacteria, and 9 were not found to be diagnosed with any organism. Past history of TB was documented in 16 patients, of which one was positive for MTB by culture and Xpert and one was positive by Xpert alone. The remaining 14 patients who tested negative for TB had one or more than one respiratory pathogens identified (Table 4).

**Table 4:** Clinical profile and bacterial aetiology of respiratory illness in TST-positive MTB-negative patients with past history of TB.

S.no	Lab number	Pathogens identified	CT value	Clinical characteristics
1	PPS 006	*H. influenzae*	17.19	Cough, fever, hilar adenopathy
		*M. catarrhalis*	29.03	
2	PPS 030	*A. flavus*	19.9	Cough, fever, tired, loss of appetite
		*H. influenzae*	39.9	
		*A. fumigatus*	36.2	
		*C. pneumoniae*	28.3	
3	PPS 031	*A. flavus*	20.5	Cough, fever, tiredness, loss of appetite, loss of weight, right lower lobe consolidation, and pleural effusion
		*P. aeruginosa*	32.18	
		*A. fumigatus*	26.83	
		*C. pneumoniae*	23.28	
		*L. pneumophila*	38.68	
		*Bordetella spp.*	23.11	
4	PPS 056	*M. catarrhalis*	31.98	Cough, difficulty in breathing
5	PPS 074	*P. aeruginosa*	30.88	Cough, fever, tiredness, difficulty in breathing, chest pain, loss of appetite, loss of weight, and massive pleural effusion
6	PPS 084	*S. pneumoniae,*	34.6	Cough, fever, tiredness, chest pain, loss of appetite, loss of weight, right lower lobe consolidation with minimal pleural effusion
		*K. pneumoniae*	38.8	
7	PPS 085	*S. pneumoniae*,	23.8	Bilateral pleural effusion
		*H. influenzae*	24.5	
8	PPS 086	*S. pneumoniae*	20.9	Fever, pleural effusion (right)
		*S. aureus*	30.23	
		*K. pneumoniae*	36.9	
		*P. aeruginosa*	36.5	
		*A. baumannii*	32.8	
9	PPS 102	No bacteria	0	Loss of appetite, abnormal chest x-ray
10	PPS 132	*P. jirovecii*	37	Cough, fever, loss of appetite, loss of weight
11	PPS 166	*S. pneumoniae*	18.8	Cough, fever, tiredness, loss of appetite
		*P. aeruginosa*	28.86	
		*A. baumannii*	34.59	
12	PPS 169	*S. pneumoniae*	35.49	Fever
		*A. baumannii*	19.4	
13	PPS 185	*S. pneumoniae*	27.82	Tired, loss of weight
14	PPS 196	*A. flavus*	35.09	Cough, fever, loss of appetite
		* *		
		*P. aeruginosa*	33.68	

## Discussion

Despite the fact that paediatric respiratory disease is one of the major contributing factors for the morbidity and mortality in children worldwide, measures taken to reduce its incidence have not intensified. There are several factors leading to this lacuna and one of them is the misdiagnosis or delayed diagnosis of TB and pneumonia. In this study, we aimed to assess whether synchronized diagnosis of TB and pneumonia in children with respiratory diseases is useful in the characterization of the disease. Our study results showed that out of 20 MTB-positive samples, 8% (5/64) were from the pneumonia group, which would have been misdiagnosed as pneumonia based on the clinician's perspective. Such misdiagnosis based on clinical characteristics have been reported elsewhere also where a case with consolidation and multicavity lesions in chest radiography was reported as bacterial pneumonia in initial diagnosis but confirmed as TB at a later stage by smear microscopy and PCR after antibiotics treatment failure (17). In another study from South Africa, Jacobs et al. reported that 32 children with the primary diagnosis of bacterial necrosis pneumonia after failure of antibiotic treatment were found to have TB per further diagnosis (18). The pneumonia group in our study included the patients that had no history of TB contacts, TST negative, no antibiotic treatment, and no previous history of TB. Diagnosis of MTB in five children from this group indicates that reliance on the risk factors alone is not enough and routine follow-up of the patients since admission is essential for appropriate TB diagnosis to avoid missing cases.

When the distribution of other bacteria was explored among the 20 MTB positives, *S. pneumoniae* was found in 9 (45%) patients. This finding is in concurrence with other studies that reported the coinfection of *S. pneumoniae* and MTB (9, 13). Such coinfections in individuals where streptococcal pneumonia is the secondary infection leads to serious health management problems than when TB alone is detected since the coinfections lead to inflammatory response in the lobe of the lungs resulting in lobular pneumonia (19). Other organisms found in MTB-positive patients with lower CT values and that could be of clinical significance were *P. jirovecii*, *K. pneumoniae,* and *H. influenzae*. All these three organisms are reported to be co-infected with MTB where the sequelae become adverse (20–22). Moreover, their clinical symptoms mimicking TB would have led to treating of the children with anti-tuberculous therapy (ATT) while missing the diagnosis of other bacterial pathogens (23). In such a scenario, although MTB may decrease with treatment, symptoms caused by other bacteria will persist, resulting in misclassification of the patients as ATT non-responders or multi drug resistant TB (MDR-TB). Similarly, if MTB diagnosis was not done in co-infected patients while other bacteria were identified, treating them with antibiotics alone will result in delayed diagnosis of MTB. Hence, simultaneous diagnosis of all the aetiological agents of respiratory disease will help in appropriate diagnosis and treatment.

Among the MTB-negative patients (165), 145 (90 from the TB group and 55 from the pneumonia group) were positive for one or more than one bacterial or fungal pathogens. In both the groups, *S. pneumoniae* was the predominantly identified organism followed by *H. influenzae.* Although a decline of 58% in pneumococcal deaths and 81% in Hib (*H. influenzae* type b disease) deaths was reported by Wahl et al. in 2019, our study findings indicate the need for continuous monitoring of these organisms’ roles, especially the serotypes involved in paediatric respiratory infections (24). Organisms that belonged to the ESKAPE group (*Enterococcus faecium*, *Staphylococcus aureus*, *Klebsiella pneumoniae*, *Acinetobacter baumannii*, *Pseudomonas aeruginosa*, *Enterobacter* spp) that are known to cause nosocomial infections were identified in our study except *Enterococcus* and *Enterobacter* spp., with different CT values (25). Increased identification of these organisms in the TB group compared with the pneumonia group is alarming since the former group comprises children with adequate antibiotic treatment. Based on the impact of antibiotic resistance emerging worldwide, WHO has listed ESKAPE pathogens under the list of bacteria for which there is urgent need of antibiotics, where vancomycin- and methicillin-resistant *S. aureus* are in the high priority group and carbapenem-resistant *A. baumannii*, *P. aeruginosa,* and *K. pneumoniae* are in the critical priority group (25, 26). Since our aim was to look at the simultaneous diagnosis of TB and the aetiology of pneumonia other than MTB, we did not focus on extensive phenotypic characterization of the bacterial spectrum identified. However, future studies focussing on parameters like antibiotic resistance profile, identification, and classification methods, will help us in obtaining better clarity on the roles of these organisms. Moreover, identification of multiple organisms in a single patient also needs further research with emphasis on limitations when a multiplex PCR panel is used for identification of bacterial spectrum (27, 28). As mentioned earlier, simultaneous extensive phenotypic characterization for genotypically identified organisms along with the patient's clinical characteristics is essential to understand the disease burden and differentiate the commensals from pathogens.

Although chest radiography is the preferred primary diagnostic test for respiratory illness, sometimes it becomes unreliable to make a definitive or differential diagnosis of pneumonia and TB (29). This is evident from our study findings where 3 out of 20 MTB positives had normal chest x-rays and 5 of them were diagnosed as pneumonia based on the radiographic findings and clinical characteristics. Similarly, among 145 patients diagnosed with other bacterial organisms, 28 (19%) had normal chest x-rays indicating the limitation of relying on chest radiography alone for empirical treatment of patients. This advocacy is supported by findings from other studies as well where 7%–21% of pneumonia patients had normal chest x-rays (30–32). In recent years, the image processing technique seems to be helpful in identifying even the minor changes that a radiologist might miss during the x-ray examination and there are several computer-aided image analysis methods involved for both pneumonia and TB diagnosis (33–35). The Verma et al. group from India have developed a computer-aided image analysis for easy identification and differentiation of TB and bacterial and viral pneumonia using the image processing technique (36). Large-scale studies based on such analysis in the near future will help in differential diagnosis of respiratory diseases for appropriate patient management.

In assessing a child with presumptive TB, especially when there is no contact history, TST serves as a useful tool because a positive TST indicates the probability of the child being infected with TB at some stage (37). This is in agreement with our study findings, where among the 48 TST-positive patients, 6 of them were MTB positives. However, we had five TST-negative patients as well among the MTB positives, emphasizing the importance of TST performance only in conjunction with other diagnostic tests. Apart from the 6 MTB positives, 33 of the 48 TST positives were diagnosed with other bacteria and 9 did not have any aetiological agent. Past history of TB was found in 7 among 33 patients, which could partially explain the colonization of other bacteria post TB disease in these 7 patients. Since there is documented evidence of post TB lung disease (PTLD) in children (38, 39), although low in number, diagnosis of bacterial pathogens in patients with past history of TB in our study warrants the necessity for continuous monitoring of the menaces and key interventions to prevent and reduce the outcome of TB on child health. For the other TST-positive children, although they did not turn out to be positive by any MTB diagnostic test and had normal chest x-rays (except one), the symptoms listed out and the presence of other bacterial pathogens implicate the need for further research. The symptoms could be either due to bacterial pathogens identified mimicking TB or MTB itself that could not be detected owing to their paucibacillary status in children (40).

### Strengths of the study

1. To our knowledge, this is the first study from India that looked at the simultaneous diagnosis of MTB and other bacterial pathogens in a paediatric population with respiratory disease.
2. Our findings indicate the need for extensive diagnostic methods to ensure that missing of the diagnoses for TB cases is avoided in the future.

### Limitations of the study

a. The smaller sample size of the study population limits us in drawing a conclusion that could lead to policy making for implementation of diagnostic methods for detection of aetiological agents other than MTB
b. We did not perform Viral PCR, which could have determined the complete aetiology of the children with respiratory symptoms who were not diagnosed with either bacteria or MTB.

## Conclusion

The results of our study suggest that TB could coexist with other respiratory pathogens causing pneumonia and an extensive diagnostic testing methods is essential for appropriate diagnosis and patient management. A large-scale prospective study from different geographical settings that uses such simultaneous detection methods for diagnosis of childhood TB and pneumonia will help in assessing the utility of these tests in rapid diagnosis of respiratory infections.

## Data Availability

The original contributions presented in the study are included in the article; further inquiries can be directed to the corresponding author.
